# Polycomb Repressive Complex 2 drives flow-sensitive endothelial states underlying vascular inflammation and disease

**DOI:** 10.1073/pnas.2603888123

**Published:** 2026-08-31

**Authors:** Divyesh Joshi, Oleksii Rukhlenko, Raja Chakraborty, Anna Tuliakova, Zhongwei Sun, Tejas Bhogale, Jessica Furtado, Hanqiang Deng, James G. Traylor, Hiroaki Imoto, Anthony Wayne Orr, George Tellides, Kathleen A. Martin, Boris N. Kholodenko, Martin A. Schwartz

**Affiliations:** ^a^ Section of Cardiovascular Medicine, Yale Cardiovascular Research Center, Department of Internal Medicine, School of Medicine, Yale University, New Haven, CT 06511; ^b^ Systems Biology Ireland, School of Medicine, University College Dublin, Dublin D04 V1W8, Ireland; ^c^ Department of Cellular and Molecular Physiology, Yale University, New Haven, CT 06520; ^d^ Department of Pathology and Translational Pathobiology, Louisiana State University Health Shreveport, Shreveport, LA 71103; ^e^ Department of Surgery, Yale University, New Haven, CT 06510; ^f^ Conway Institute of Biomolecular and Biomedical Research, University College Dublin, Dublin D04 V1W8, Ireland; ^g^ Department of Cell Biology, Yale University, New Haven, CT 06510; ^h^ Department of Biomedical Engineering, Yale University, New Haven, CT 06510

**Keywords:** vascular inflammation, atherosclerosis, PRC2, fluid shear stress, EZH2

## Abstract

Atherosclerotic cardiovascular disease (ASCVD), the leading cause of mortality worldwide, is driven by endothelial cell (EC) inflammatory activation and counterbalanced by protective mechanosensitive transcription factors Klf2 and Klf4 (Klf2/4). Plaques initiate in arterial curves or branches where disturbed shear stress (DSS) restrains Klf2/4 expression and promotes inflammatory activation of the endothelium. Importantly, DSS determines plaque vulnerability to rupture, the event that precipitates heart attack or stroke in advanced disease. However, the central determinants of endothelial inflammatory mechanotransduction and their interventional targeting to treat ASCVD pathology remain underexplored. Here, we identify Polycomb Repressive Complex (PRC) 2 as a potent, therapeutically targetable driver of vascular endothelial inflammation and ASCVD progression. PRC2 trimethylates Histone H3 Lysine27 in gene promoters/enhancers, including Klf2/4, to block gene transcription. Integrated mechanistic computation and experimental studies identified PRC2 as a central determinant of the EC state transition to inflammatory activation. PRC2 activity is elevated in endothelium from human ASCVD lesions. In murine models of acute and chronic vascular inflammation, interventional treatment with tazemetostat, a Federal Drug Administration (FDA)-approved inhibitor of the PRC2 methyltransferase EZH2, reduced endothelial inflammatory genes, slowed disease progression, and drastically improved markers of plaque stability. This study elucidates a fundamental epigenetic mechanism in vascular inflammation and suggests a potential treatment for advanced and chronic cardiovascular inflammatory diseases.

## Significance

Vascular inflammation, the cause of atherosclerosis and acute lung injury among other cardiovascular disorders, is the major undertreated cause of disability and death worldwide. Here, we report that the epigenetic transcriptional repressor, the Polycomb Repressive Complex 2 (PRC2) is a critical node that suppresses anti-inflammatory mediators and promotes inflammatory gene expression in vascular endothelial cells. PRC2 is activated in inflamed endothelium in humans and mice, and its small molecule inhibition strongly improves outcomes in preclinical mouse models of acute lung injury and atherosclerosis. This study identifies PRC2 as a therapeutic target in cardiovascular disease and suggests PRC2 inhibitors for clinical use.

Atherosclerotic cardiovascular disease (ASCVD), the progressive inflammatory disease of arteries that leads to heart attack or stroke, is the most frequent cause of mortality worldwide. Plaques arise and progress as a consequence of converging metabolic, inflammatory, and biomechanical risk factors (1, 2). Vascular endothelial cells (ECs) are central to this process, with EC dysfunction being the earliest detectable event in plaque initiation. EC inflammatory activation leads to recruitment of leukocytes and smooth muscle cells that form the bulk of the plaque. While most atherosclerotic lesions are clinically silent, their progression to vulnerable plaques, characterized by thin fibrous caps, large necrotic cores, and heightened inflammation can lead to plaque rupture and thrombosis. Understanding the molecular mechanisms that drive the transitions between stable and unstable plaque is critical for developing new therapeutics.

ECs lining the vasculature sense and transduce fluid shear stress (FSS) from blood flow into biochemical responses that ultimately control gene transcription and thus cell phenotypes representing different cell states. Whereas cell states were initially defined solely by observable phenotypes, they can now be characterized by omics measurements, e.g., transcriptomics that describe genome-wide expression levels. In stable or metastable states, these omics patterns are reproducible, reflecting the underlying regulatory networks that maintain particular cell states. Uncovering the genotype–phenotype links requires understanding the causal network connections between key regulatory genes and how they influence one another and the resulting EC phenotype. In other words, we must uncover the regulatory networks that control EC behavior and their cell fate decisions.

We recently presented the cell State Transition Assessment and Regulation (cSTAR) approach, which takes omics data as an input to map cell states, models transitions between them, and deciphers the regulatory networks that control cell fate decisions and state transitions (3). Applying this approach to assess EC transcriptomic responses under different FSS conditions distinguished EC states corresponding to physiological shear stress (PSS), pathologically high (HSS) or low (LSS), and disturbed FSS (DSS) and identified cell cycle as a key regulator of flow-dependent vessel remodeling (4). Further, unbiased regulatory analysis predicted a strong epigenetic contribution from the Polycomb Repressive Complex 2 (PRC2)-dependent network, identifying it as a key vessel destabilization module that governs EC trajectories and state transitions under different FSS profiles.

**Fig. 1.**
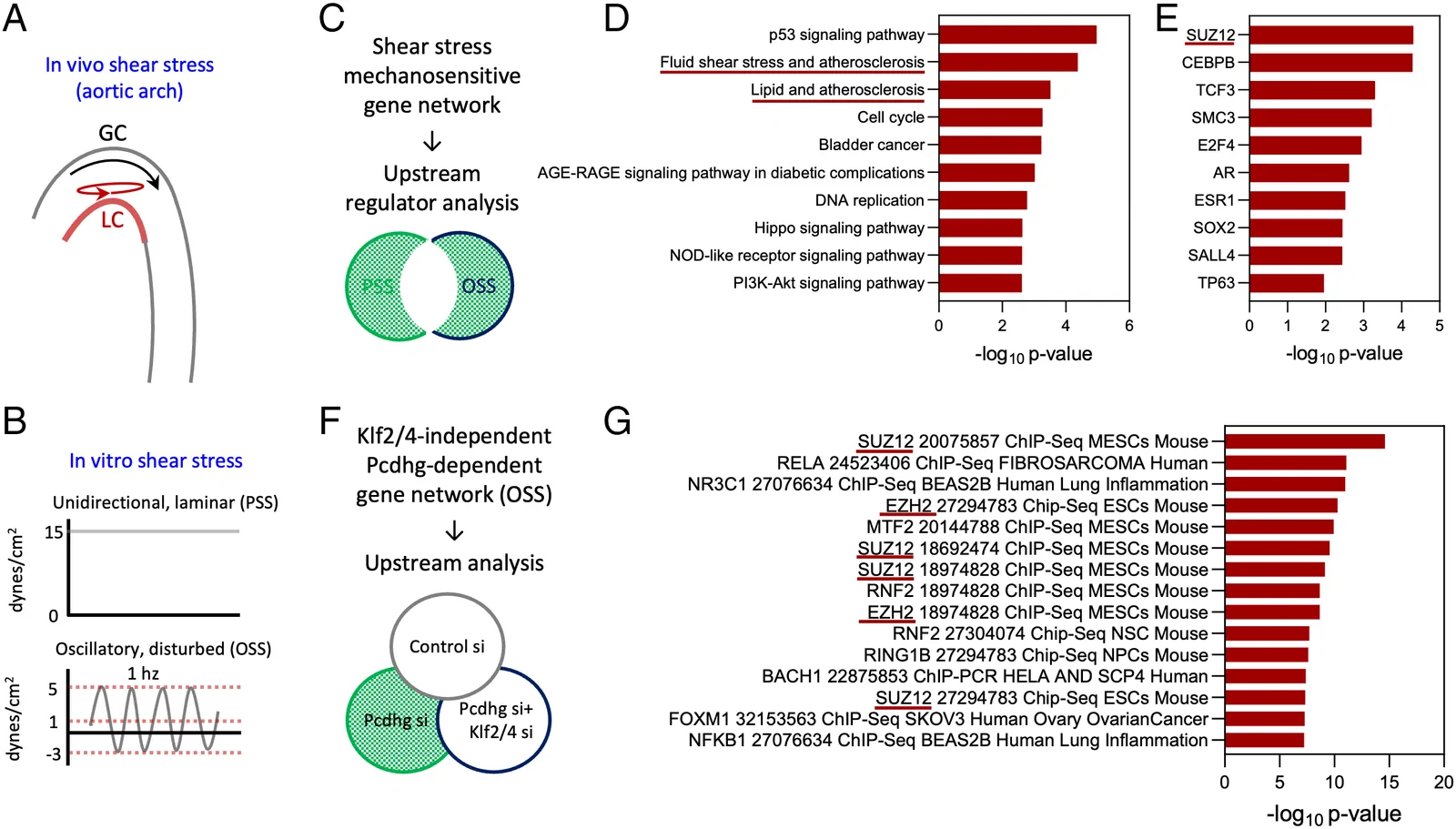
PRC2 governs EC gene expression in response to inflammatory flow. (*A* and *B*) Schematic of the aortic arch region showing the greater curvature (GC) under PSS and the lesser curvature (LC) under DSS (*A*), and in vitro modeling of PSS and OSS respectively (*B*). (*C*) Bulk RNAseq analysis of HUVECs under PSS or OSS, followed by EnrichR analysis of DEGs (green shaded region) to identify enriched pathways/processes (*D*) and common upstream regulators of the DEGs between PSS and OSS (*E*). (*F* and *G*) Bulk RNAseq analysis of Control si, Pcdhg si and Pcdhg + Klf2/4 triple si HUVECs exposed to OSS to identify Pcdhg-dependent, Klf2/4 independent DEGs (green shaded region) (*F*). These DEGs were then analyzed using EnrichR to identify common upstream regulators (*G*).

FSS mechanosensing by the vascular endothelium is a major determinant of the sites of initiation, progression, and transition of atherosclerotic plaques to the rupture-prone vulnerable phenotype (5). Protective physiological FSS (PSS) in straight regions of arteries upregulates Kruppel like factors 2 and 4 (Klf2/4), which promote anti-inflammatory, atheroprotective gene expression (Fig. 1*A*) (6). EC Klf2 and Klf4 are coregulated homologous genes with substantial functional redundancy and overlapping gene targets. In contrast, vessel branches or curves typically develop regions with disturbed shear stress (DSS), commonly modeled in vitro using oscillatory shear stress (OSS), where Klf2/4 expression is low and pro-inflammatory NF-κB is activated (1, 2). Klf2/4 and NF-κB are mutually inhibitory, with the balance between the two pathways playing a crucial role in disease susceptibility and progression (7–11). Consistent with their protective function, increasing or decreasing EC Klf2/4 expression reduces or exacerbates, respectively, the incidence and severity of vascular inflammation and disease in multiple preclinical models (12, 13). Here too, our limited understanding of shear stress-dependent Klf2/4 transcriptional regulation has hindered the development of clinically effective strategies to elevate Klf2/4 for ASCVD treatment.

To address this gap, we carried out computational analyses of published transcriptomic data to identify candidate genes and pathways that suppress Kl2/4 and promote endothelial inflammatory activation. These analyses identified PRC2 and its core subunits as the most prominent group of mediators. PRC2 is best known for its role in silencing developmental and oncogenic genes via Histone H3K27 trimethylation (H3K27me3) (14, 15). It is the only known epigenetic writer of H3K27me3, with thousands of known gene targets including Klf2/4, many of which are important for ASCVD (16–19). Multiple studies have explored its contribution to general and vascular inflammation that is associated with diverse CVDs (20–22). Consequently, blocking PRC2 function by deletion of a core component in ECs, monocytes, macrophages or T-cells, or by inhibition of the major methyltransferase EZH2, reduced vascular inflammatory diseases and atherogenesis (22–28). EZH2 inhibitors, including GSK126, DZNep, and Tazemetostat (EPZ-6438 or Tz), showed promise in preventing vascular inflammatory diseases (16, 29–31).

Our study builds on this established role of PRC2/EZH2 in ASCVD, both exploring mechanisms and providing proof-of-concept for clinical use. We report that three independent computational analyses of endothelial inflammatory mechanotransduction converged on PRC2 as a central regulator of inflammatory cell state and state transitions. Second, unlike preventative experimental design in previous studies, we adopted a clinically relevant interventional regime of PRC2/EZH2 inhibition in established disease and focused on plaque phenotype as the principal end point. This strategy was based on the key role of plaque propensity to rupture or erode rather than plaque size in clinical events. Tz was chosen for these studies due to its excellent safety profile and FDA approval for long term treatment (ClinicalTrials.gov ID NCT02875548). Building on previous work that identified a function for PRC2 in endothelial inflammation and atherosclerosis (20, 26, 28, 30), we now report a key role for PRC2/EZH2 in responses to diverse mechanical and biochemical stresses and atherosclerotic plaque vulnerability, thus establishing a critical mechanistic link between epigenetic repression and CVD pathogenesis. These insights advance Tz for potential treatment of high-risk patients with atherosclerosis and other inflammatory vascular disorders.

## Results

### PRC2 Governs EC Gene Expression in Response to Inflammatory Flow.

To identify relevant candidates that control EC inflammatory gene expression, we first examined gene expression in ECs under protective PSS vs. athero-promoting OSS using our previously published RNAseq data (4) (Fig. 1 *B* and *C*). Analysis of differentially expressed genes (DEGs) showed the expected elevation of Klf2, Klf4, and their target genes Nos3 (eNOS) and Thbd (Thrombomodulin) and antioxidant response factor Nfe2l2 (Nrf2) under PSS while the inflammatory mediators Ccl2 (Mcp-1), Vcam1, and E-selectin (Sele) were elevated under OSS, confirming expected responses (*SI Appendix*, Fig. S1*A* and Dataset S1*A*: Shear-dep DEGs). Pathway/process analysis of DEGs showed overrepresentation of FSS and atherosclerosis categories (Fig. 1*D*), supporting biological relevance. To identify transcriptional regulators that mediate these changes, we assessed upstream regulators using EnrichR (32), which identified the core PRC2 component SUZ12 as the top hit (Fig. 1*E*) and the PRC2 catalytic subunit EZH2 as an additional hit (Dataset S1*B*: TFs_Shear-dep DEGs, hit #62). This analysis suggests PRC2 as a key node governing EC response to inflammatory FSS.

We recently reported that the protocadherin gamma gene cluster (Pcdhg) in ECs potently suppresses Klf2/4, thus, is a component of the proinflammatory cascade in atherosclerosis (13). As an independent approach to identify relevant regulatory pathways, we assessed effects of Pcdhg si (Fig. 1*F*). This experiment included Pcdhg si + Klf2/4 si to distinguish direct Pcdhg targets from those affected through Klf2/4. EnrichR analysis of upstream regulators of the Pcdhg-dependent, Klf2/4-independent DEGs (Dataset S1*C*: TFs_Pcdhg-dep DEGs) again identified EZH2 and SUZ12 as two of the most significant hits (Fig. 1*G* and Dataset S1*D*: TFs_Pcdhg-dep DEGs). Of the 494 DEGs (311 up and 183 down after Pcdhg suppression), 161 were identified as PRC2 targets, two-thirds (110/161) of which were upregulated after Pcdhg si while one-third (51/161) were downregulated (Dataset S1*C*: Pcdhg-dep DEGs). As PRC2 is mainly a transcriptional repressor, these data indicate that Pcdhg activates PRC2. This analysis also identified NF-κB, a known inhibitor of Klf2/4, and the PRC1 component RING1B, further validating the approach (Fig. 1*G*). Analysis of these DEGs using X2KWeb (32), another tool for upstream regulator analysis, similarly showed PRC2 as a principal mediator of Pcdhg function (*SI Appendix*, Fig. S1*B*). Additionally, our original genome-wide CRISPR screen (13) identified the PRC2 component Jarid2 as a suppressor of Klf2, providing further support. Although expression of PRC2 components themselves were unaffected by Pcdhg si (*SI Appendix*, Fig. S1*C*), ruling out a transcriptional feedback loop, EZH2 and Suz12 proteins were reduced (concomitant with the increase in Klf4) upon Pcdhg si, suggesting an effect on PRC2 complex stability (33) (*SI Appendix*, Fig. S1 *D* and *E*). Three independent approaches thus identify PRC2 as a regulator of EC inflammatory gene expression.

### cSTAR Identifies PRC2 as a Key Mediator of OSS State.

**Fig. 2.**
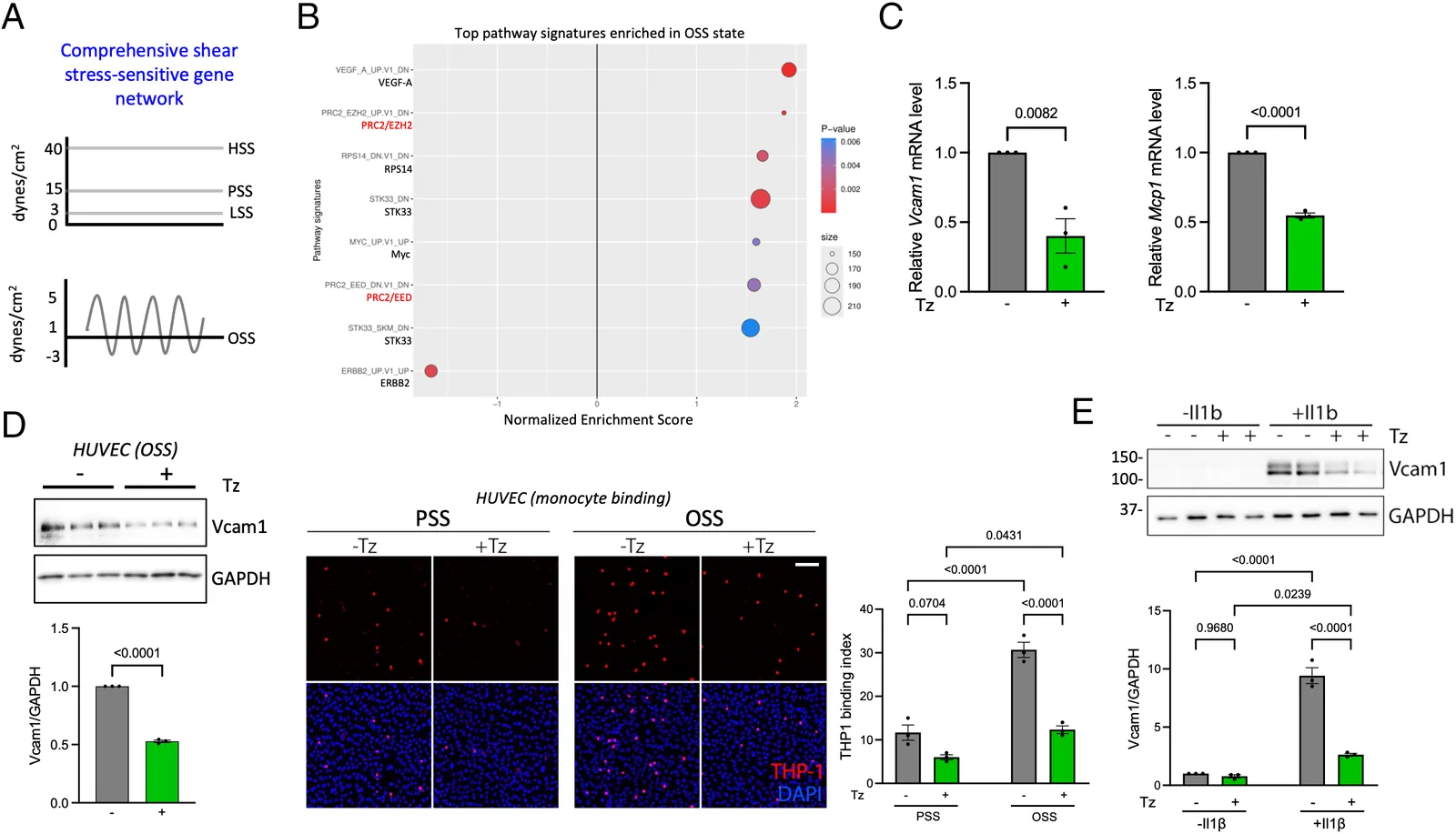
cSTAR identifies PRC2 as a key mediator of the OSS state. (*A*) HUVECs were exposed to the indicated shear stress profiles and assessed by bulk RNA sequencing. Data were analyzed using cSTAR, and the DPD scores were calculated. (*B*) Components of the STV_OSS_ were analyzed using GSEA (C6 collection). Top upregulated pathways are presented. Size of the circles indicates number of genes, while color represents the FDR adjusted *P*-value. (*C*) qPCR for pro-inflammatory adhesion receptor *Vcam1* and chemokine *MCP1* in HUVECs under OSS for 16 h with or without Tz (N = 3 independent experiments). (*D*) Immunoblot for VCAM1 under OSS (*Left*) and THP-1 monocyte binding to HUVECs under PSS or OSS for 16 h with or without Tz (N = 3 independent experiments). Graphs: quantitation of VCAM1 normalized to GAPDH (*Left*) and THP1 monocytes per field per condition (THP1 binding index) (*Right*). (*E*) Immunoblot for VCAM1 in HUVECs, untreated or treated with Il1β (1 ng/mL) for 16 h with or without Tz (N = 3 independent experiments). Graph: quantitation of VCAM1 normalized to GAPDH. Values are means ± SEM. Statistical analysis used unpaired two-tailed Student’s *t* test (*C*) or one-way ANOVA (*D* and *E*). [Scale bar, (*D*) 100 μm.]

To gain deeper insights into the regulatory networks that govern EC inflammatory gene expression, we turned to cSTAR, which we recently used to identify different EC states and state transitions related to flow-dependent vessel remodeling (4). Our analysis used RNAseq data from HUVECs exposed to a comprehensive repertoire of shear stress profiles to determine their differential transcriptional states and identify key determinants of artery stabilization, remodeling, and disease susceptibility (Fig. 2*A*). These shear stress profiles were based on the set point model (34) where PSS that is at or near the set point stabilizes vessels to prevent remodeling; low shear stress (LSS) that is below the set point promotes inward artery remodeling; high shear stress (HSS) above the set point promotes outward remodeling; and OSS sensitizes ECs to other risk factors to promote atherosclerosis. Cells under no-flow, static conditions (STAT) were included as a control. To map distinct EC transcriptomic states, we applied machine learning using support vector machines (SVMs) to identify hyperplanes that separate different transcriptomic patterns. In the transcriptomic space, the shortest paths for transitions between these EC states are represented by State Transition Vectors (STVs), which are unit vectors normal to the hyperplanes separating EC states. In this analysis, we distinguished the following EC states: i) those observed under LSS vs. HSS, with the corresponding STV denoted as STV_FSS_; ii) stable EC states under PSS vs. those associated with inward (LSS) or outward (HSS) vessel remodeling, denoted as STV_remod_; and iii) EC states under OSS associated with pathological remodeling or atherosclerosis, denoted as STV_OSS._ All three STVs appear to be orthogonal in the transcriptomic space, suggesting distinct transcriptomic and phenotypic EC responses to perturbations along their respective axes.

Changes in phenotypic EC features along transition trajectories between EC states are quantified using Dynamic Phenotype Descriptors (DPDs), calculated along each STV (4). The DPD score of a given EC state is defined as the distance from this state to the separating hyperplane with the positive or negative sign determined by the direction of the STV (3). While the STV_FSS_, STV_remod_, and STV_OSS_ identify key genes distinguishing EC states, changes in the corresponding DPD_FSS_, DPD_remod_, and DPD_OSS_ following perturbations of these regulatory genes capture their effects on EC phenotypes. Applying gene set enrichment analysis (GSEA) to the STV_OSS_ component genes in this published dataset, we identified activity of PRC2 components as significant contributors to the regulation of specifically OSS state (Fig. 2*B*).

### Experimental Verification by Pharmacological Inhibition of PRC2/EZH2.

These computational results prompted us to experimentally test the role of PRC2. PRC2 comprises methyltransferases EZH1/EZH2 along with other core and accessory components. EZH2 has much higher catalytic activity than EZH1, is expressed at higher levels in ECs (*SI Appendix*, Fig. S2 *A* and *B*) and its depletion strongly reduces H3K27me3 (*SI Appendix*, Fig. S2*C*), confirming it as the major H3K27 methyltransferase in ECs. EZH2 can be targeted by Tazemetostat (Tz), (also called EPZ-6438) an FDA-approved drug for cancer therapy (35) that specifically inhibits EZH2 catalytic activity (35-fold stronger inhibition of EZH2 than EZH1, and ~4,500 fold stronger than other methyltransferases). ECs exposed to inflammatory OSS in the presence of Tz showed reduced expression of *Vcam1*, a receptor that mediates leukocyte recruitment to inflamed vessels, and *Mcp1/Ccl2*, an inflammatory chemokine involved in monocyte recruitment (Fig. 2*C*). Vcam1 protein levels were similarly reduced, as was binding of THP-1 monocytes to ECs activated by OSS (Fig. 2*D*). Tz similarly reduced expression of the leukocyte recruitment gene Vcam1 on ECs exposed to the inflammatory cytokine Il1β, or the bacterial lipopolysaccharide (LPS) (Fig. 2*E* and *SI Appendix*, Fig. S2*D*).

### PRC2 Promotes Multiple Pro-Atherogenic Pathways.

**Fig. 3.**
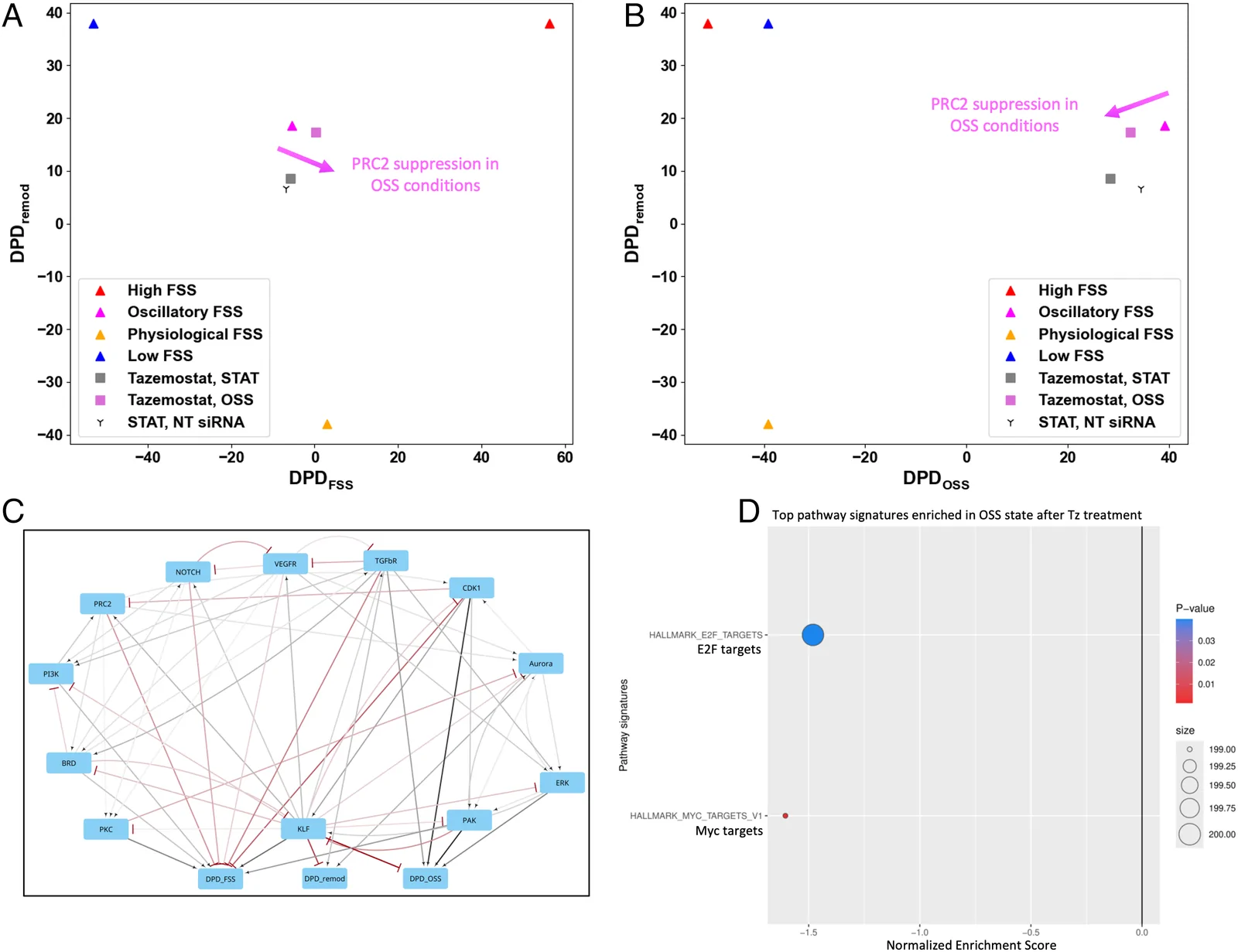
Experimental verification by pharmacological inhibition of PRC2/EZH2. (*A* and *B*) 2D-projections of the 3D space of DPDs that assess HUVEC phenotype. HUVECs were exposed to the indicated shear stress profiles, with or without Tazemetostat (Tz) treatment, and assessed by bulk RNA sequencing. Data were analyzed using cSTAR, and the 3 DPD scores were calculated. (*C*) The cSTAR-inferred network presents causal connections between signaling modules and their connections to the phenotypic modules (DPD_FSS_, DPD_remod_, and DPD_OSS_). Arrowheads indicate activation, and blunt ends indicate inhibition. Intensity of the line color indicates the absolute values of interaction strengths. (*D*) GSEA analysis of the effect of Tz treatment in OSS condition confirms the network-based prediction that PRC2 inhibition suppresses the cell cycle. Color of the circles reflects the FDR adjusted *P*-value.

We next did RNAseq on ECs treated with or without Tz and used cSTAR to integrate these data with our previous cSTAR pathway analysis. These results showed that Tz modesty but significantly reduced the pathological OSS score, DPD_OSS,_ whose increase is associated with the atherosclerosis-prone state (Fig. 3 *A* and *B*). Additionally, Tz decreased the remodeling score, DPD_remod_, and increased the FSS score, DPD_FSS_; these changes move cell states in the direction of PSS state, i.e., normal physiological state of ECs. These data reinforce PRC2 as a component of the inflammatory pathways in ECs that promote pathological remodeling and disease.

To infer the regulatory network that governs EC inflammatory vs. atheroprotective states, we integrated LINCS transcriptomic HUVEC perturbation data (36) with published datasets on NOTCH (37, 38) and KLF2/4 (39, 40) perturbations, chosen for these proteins’ critical roles in arterial identity and atherosclerosis. We then applied cSTAR to infer causal links between signaling modules and their impact on the three DPD scores (*Materials and Methods*). The inferred causal connections between protein modules (Fig. 3*C*) and their immediate network-predicted influences on the DPD modules strongly suggests a key role of PRC2 in the regulation of EC progression through the cell cycle and EC responses to FSS (Dataset S2).

The inferred connections suggest that PRC2 is mainly activated downstream of PI3K, and subsequently activates CDK1 and Aurora kinases, promoting cell cycle progression, a known feature of the OSS state (4, 41). Notch suppresses the VEGFR module, which leads to suppression of Aurora kinase and, therefore cell cycle progression. Our previous work showed that cell cycle progression correlates with the inflammatory response to OSS (4). Of particular interest, ECs in a late G1 arrested state had active CDK2, which suppressed Smad2/3 signaling that is associated with both inward artery remodeling under low shear and inflammatory activation under OSS. Our inferred network also predicted that PRC2 activates BET-bromodomain (BRD) proteins, which in turn activates the pro-atherogenic TGFβ pathway and suppresses expression of KLF2/4 (Dataset S2). Analysis of DEGs (Dataset S2*D*: Klf family DEGs_OSS vs. Tz + OSS) shows that Tz in OSS conditions significantly upregulates KLF2, −3, −4, −6, −7, −9, −10, −11, and −13. PRC2 is thus predicted to exert pro-atherogenic effects via multiple mechanisms.

Using cSTAR, we predicted cell-wide effects of increasing the activities of VEGFR, PAK, ERK, CDK1, Aurora kinase, BRD proteins, and TGFβ pathway on the DPD_OSS_ score (*Materials and Methods*), suggesting that activation of each of these modules also increases inflammatory responses (Dataset S2). Two immediate suppressors of KLF2/4 expression in the inferred network are the PAK and BRD modules. VEGFR, CDK1, ERK, PAK, and Aurora kinase form a coactivation cluster, which drives cell cycle progression by activation of CDK1 and Aurora kinase, and suppresses KLF2/4 expression via PAK. In contrast, BRD and TGFβ pathways form a distinct coactivation cluster that does not substantially impact the cell cycle but suppresses KLF2/4 expression and promotes other pro-inflammatory changes mediated by TGFβ signaling. As mentioned above, PRC2 stimulates both of these pro-inflammatory clusters. In addition to network analysis, we analyzed transcriptome changes induced by PRC2 inhibition using the GSEA algorithm, which confirms the effect of PRC2 on cell cycle (Fig. 3*D*).

### PRC2 Regulates the Klf2/4 Pathway by Both Cell-Cycle-Dependent and Independent Mechanisms.

**Fig. 4.**
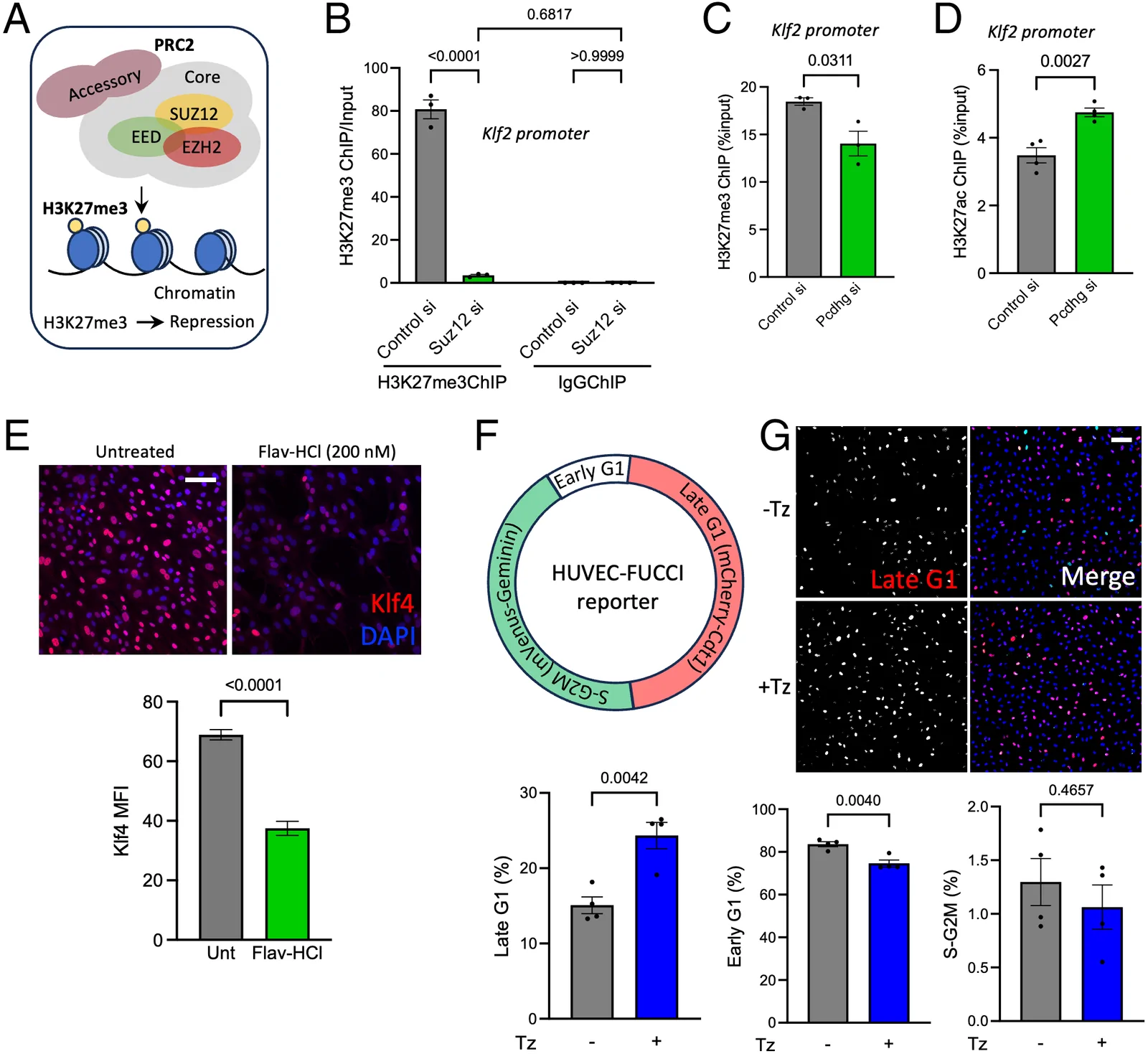
PRC2 promotes multiple pro-atherogenic pathways. (*A*) Schematic of PRC2 showing components and function. (*B*) H3K27me3 ChIP-qPCR on the Klf2 promoter in HUVECs. Depletion of PRC2 by Suz12 si was used as an internal control to confirm PRC2 specificity of H3K27me3 (N = 3 independent experiments). (*C* and *D*) H3K27me3 and H3K27ac ChIP-qPCR analysis of Control si and Pcdhg si HUVECs on the Klf2 promoter (N = 3 independent experiments). (*E*) HUVECs under PSS were incubated for 24 h with or without Flavopiridol (Flav-HCl) and nuclear Klf4 MFI was quantified (N > 500 cells). (*F*) Schematic of FUCCI reporter system. (*G*) FUCCI-HUVECs were incubated for 24 h with or without Tz and the percentage of cells in different cell cycle states quantified (N = 4 independent experiments, n > 3,000 cells/experiment). Values are means ± SEM. Statistical analysis used one-way ANOVA (*B*) or unpaired two-tailed Student’s *t* test (*C*–*E* and *G*). [Scale bar, (*E* and *G*) 100 μm.]

PRC2 is a protein complex comprised of core EZH2, SUZ12, EED, and RbAp46/48 subunits plus accessory factors. EZH2 is the catalytic subunit of PRC2, which methylates lysine 27 on Histone H3 (H3K27me3) in promoters and enhancers of target genes to maintain transcriptional repression (Fig. 4*A*). Previous studies characterizing PRC2-regulated genes (22, 42), plus data from Fig. 2, suggested that Klf2 and 4 could be relevant gene targets. ChIP-qPCR with an antibody to H3K27me3 pulled down the Klf2 promoter, whereas control IgG did not (Fig. 4*B*), confirming that Klf2 is a direct target in HUVECs under the conditions tested. This signal was lost upon Suz12 si, demonstrating a requirement for PRC2. Analysis of H3K27me3 ChIPseq datasets in HUVECs from the UCSC Genome Browser also showed peaks for H3K27me3 on the Klf2/4 promoters (marked by nearby peaks for H3K27ac) (*SI Appendix*, Fig. S3*A*). As expected, ChIP-seq with an antibody to H3K27ac, an activating mark that is mutually exclusive with H3K27me3 on the same histone, showed that Pcdhg si significantly increased H3K27ac at the Klf2 promoter (*SI Appendix*, Fig. S3*B*). ChIP-qPCR confirmed that Pcdhg si decreased H3K27me3 and increased H3K27ac on the Klf2 promoter (Fig. 4 *C* and *D*). Under basal, static conditions in culture, Klf2 is modestly expressed while Klf4 is very low, which could explain why we did not observe a significant increase in H3K27ac mark on the Klf4 promoter upon Pcdhg si. In addition to this direct regulation of Klf2/4 promoters, PRC2/EZH2 suppresses endothelial ERK5, the kinase essential for flow-induced Klf2/4 expression (26). However, we did not observe a change in ERK5 activation upon Pcdhg si. Pcdhg suppression of Klf2/4 thus correlates with PRC2 epigenetic modification of their promoters.

We next turned to cSTAR to assess this connection. cSTAR analysis of transcriptome changes induced by PRC2 inhibition using the GSEA algorithm predicts that PRC2 suppresses Klf2/4 expression via two distinct modules, one dependent on and the other independent of cell cycle (Fig. 3 *C* and *D*). To test the role of cell cycle on the Klf2/4 pathway, we used a chemical inhibitor, Flavopiridol, that targets a number of CDKs including CDKs 1, 2, 4, 6, 9. CDK inhibition with Flavopiridol (200 nM) reduced PSS-induced Klf4 levels by ~50% (Fig. 4*E*), supporting the cSTAR prediction that cell cycle state regulates Klf2/4 levels. In the cardiovascular system, PSS promotes not only Klf2/4, but also arterial identity and stability, which is associated with a late G1 arrested state (4, 43). To evaluate the role of PRC2/EZH2 in cell cycle regulation, we used the Fluorescent Ubiquitination-based Cell Cycle Indicator (FUCCI) system, as we did previously (4). FUCCI distinguishes cells in early G_1_, late G_1_, and S-G_2_M stages based on the expression of the fusion proteins mCherry-Cdt1 and mVenus-Geminin that are ubiquitinated and degraded in different stages of the cell cycle (Fig. 4*F*). Stable FUCCI-expressing HUVECs and HAECs were scored for percent unlabeled (Early G_1_), red (Late G_1_), or green (S-G_2_M). Treating cells with Tz for 24 h increased the late G_1_ arrested state and decreased early G1, with a trend toward fewer cells in S-G2-M (Fig. 4*G* and *SI Appendix*, Fig. S3*C*). These results thus confirm a key cSTAR prediction.

### Elevated PRC2 Activity in Atherosclerotic CVD Suppresses Protective Klf2/4.

**Fig. 5.**
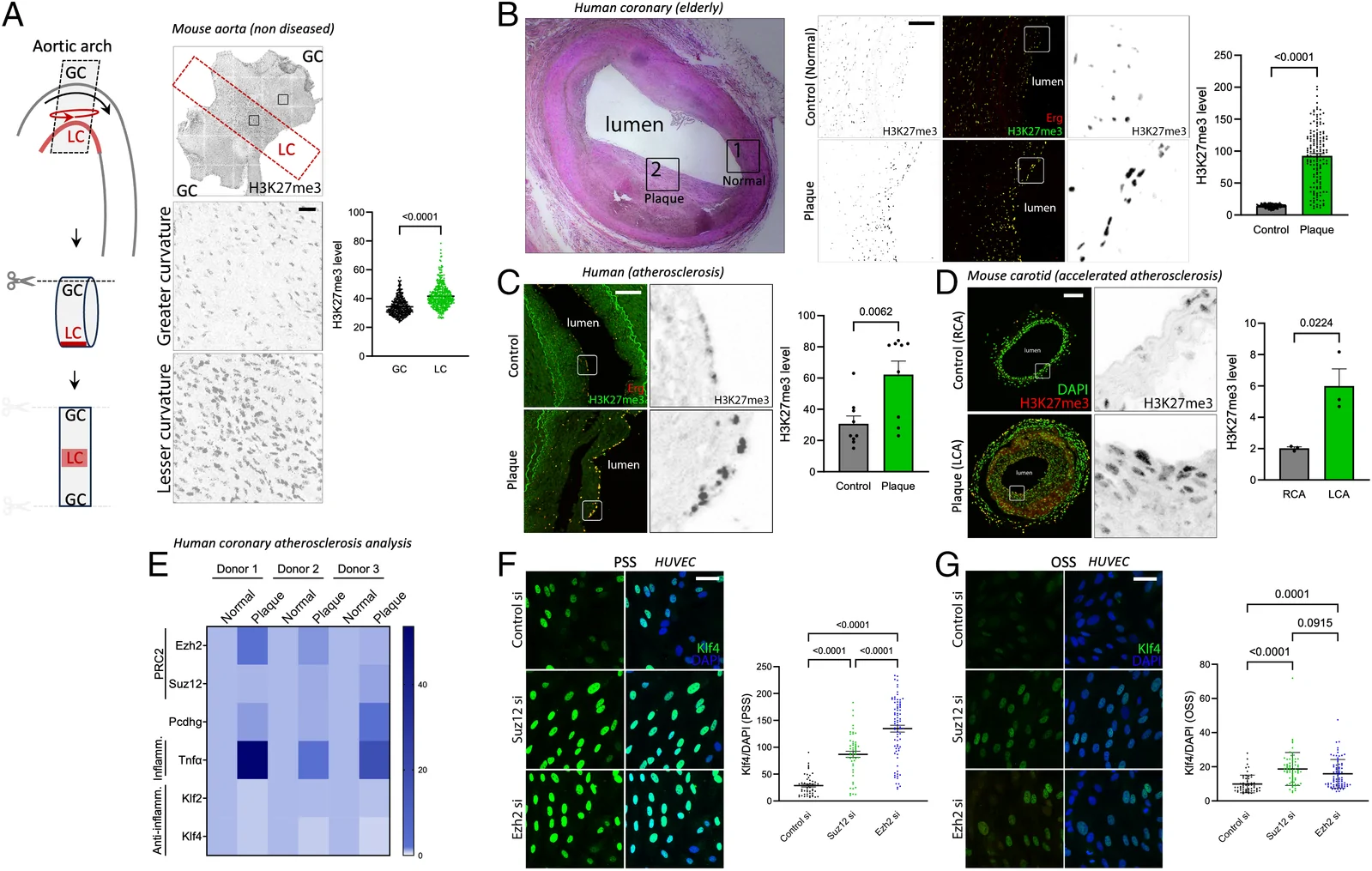
Elevated PRC2 activity in atherosclerotic CVD suppresses protective Klf2/4. (*A*) Aortic arch region prepared en face and immunostained for H3K27me3 as a marker for PRC2 activity. GC is under PSS, LC is under DSS as shown in the schematic (N = 3 male animals). Graph: quantitation of H3K27me3 MFI. (*B*) Human coronary artery sections from deidentified elderly donors stained with H&E (*Left*) and H3K27me3 levels (*Right*), comparing regions of plaque (Plaque) to the adjacent regions of the same artery without evident plaque (Normal). Sections were counterstained with Erg to mark ECs (N = 3 donors). Graph: quantitation of H3K27me3 MFI in ECs. (*C*) Artery sections from deidentified human CVD patients containing atherosclerotic plaques and healthy controls stained with H3K27me3. Sections were counterstained with Erg to mark ECs (N = 8 donors). Graph: quantitation of H3K27me3 MFI in ECs. (*D*) Carotids from mouse Partial Carotid Artery (PCA) Ligation model of accelerated atherosclerosis stained for H3K27me3. Sections were counterstained with DAPI to mark nuclei (N = 3 male animals). RCA: Right Carotid Artery (control), LCA: Left Carotid Artery (plaque). Graph: quantitation of H3K27me3 MFI. (*E*) Reanalysis of Ezh2, Suz12, and Pcdhg in ECs from published scRNAseq data from deidentified human ASCVD patients comparing regions of plaque (Plaque) to an adjacent plaque-free region of the same artery (Normal) (45). TNFα and Klf2/4 were used as internal controls for plaque ECs and normal ECs respectively (N = 3 donors). (*F* and *G*) Depletion of PRC2 by Suz12 si or Ezh2 si in HUVECs exposed to PSS (*F*) or OSS (*G*) and stained for Klf4 (N = 3 independent experiments). Graph: quantitation of Klf4 MFI. Values are means ± SEM. Statistical analysis used unpaired two-tailed Student’s *t* test (*A*–*D*) or one-way ANOVA (*F* and *G*). [Scale bar, (*A*) 50 μm, (*B*–*D*) 100 μm, (*F* and *G*) 20 μm.]

We next examined mouse and human arteries to address epigenetic H3K27 modification in vivo. In mice, the aortic arch contains adjacent regions under protective PSS (GC) and disease-prone DSS (LC) where the endothelium expresses inflammatory molecules such as VCAM1 and ICAM1 (44). En face staining of this tissue revealed higher H3K27me3 in the LC relative to the GC (Fig. 5*A*), positively correlating PRC2 activity with disease susceptibility.

Human coronary arteries from deidentified elderly donors without symptomatic ASCVD showed ~4× higher H3K27me3 staining in plaque ECs compared to adjacent unaffected regions of the same arteries (Fig. 5*B*). Arteries from deidentified human ASCVD patients also showed a ~3× increase in intimal H3K27me3 compared to control healthy donors without disease (Fig. 5*C*). Plaques from a mouse model of ASCVD (PCA Ligation and HFD for 3 wk) again showed ~3× higher H3K27me3 staining (Fig. 5*D*). H3K27me3 signal localized to both luminal ECs and to cells deeper in the vessel wall that were positive for lymphocyte common antigen, CD45 (*SI Appendix*, Fig. S4*A*). H3K27me3 is thus elevated in multiple instances of ASCVD, in ECs and leukocytes in the vessel wall.

Examination of published scRNAseq datasets from human atherosclerotic plaques also showed increased EZH2, SUZ12, and Pcdhg expression in plaque endothelium, compared to the control adjacent region of the same artery (45) (Fig. 5*E*). TNFα levels correlated positively, while Klf2 and Klf4 levels correlated negatively with ASCVD, serving as internal inflammatory and anti-inflammatory controls, respectively. This trend was less obvious in immune cells and SMCs (*SI Appendix*, Fig. S4*B*). Thus, tissue staining and published scRNAseq data confirm upregulation/activation of PRC2 in plaque endothelium and perhaps other cell types. Interestingly, we observed an inverse correlation between the expression of arterial marker Efnb2 and the inflammatory markers Mmp2, Selp, and Ccl2 in plaque ECs (*SI Appendix*, Fig. S4 *C* and *D*) supporting the notion that arterial resistance to atherosclerotic remodeling indicates an anti-inflammatory state, and that PRC2 suppresses this state.

To directly test whether PRC2 regulates Klf2/4 under flow, ECs in vitro transfected with control, Suz12, or Ezh2 siRNA were examined under PSS and OSS. We assessed Klf4 by immunostaining (due to unavailability of high-quality antibodies for Klf2). Suz12 si and Ezh2 si elevated Klf4 in ECs under both flow patterns (Fig. 5 *F* and *G*). Pharmacological inhibition with Tz similarly elevated Klf4 in ECs under PSS (*SI Appendix*, Fig. S5*A*). We obtained similar results with two additional inhibitors of PRC2, namely GSK343 and Tulmimetostat, in both HUVECs and HAECs, showing functional specificity and robustness (*SI Appendix*, Fig. S5 *B*–*D*). PRC2 thus suppresses protective Klf2/4 in ECs, consistent with H3K27me3 marks on the promoter/enhancer regions of these genes.

### Tz Ameliorates Endothelial Activation and Lung Injury.

**Fig. 6.**
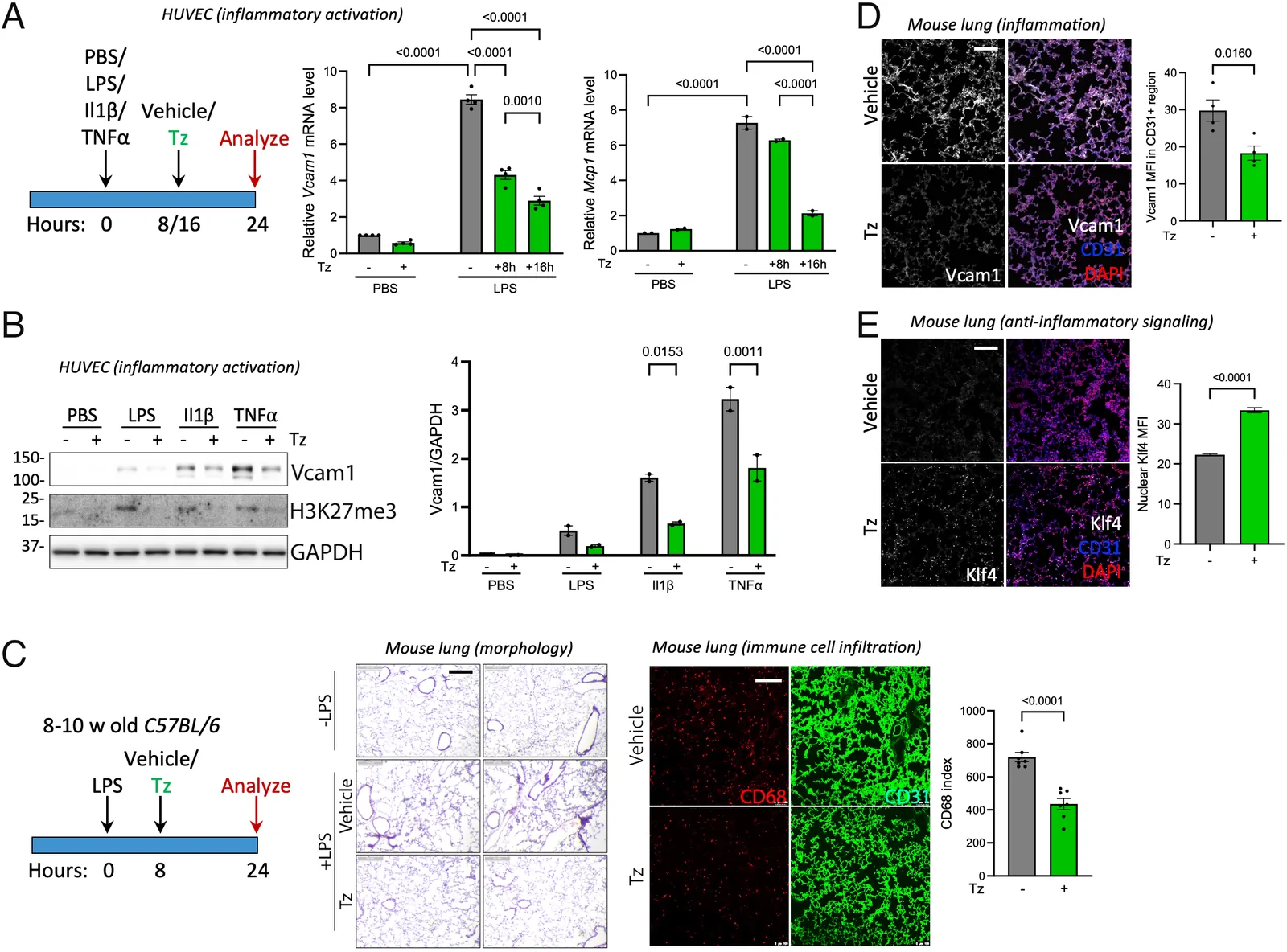
Tz ameliorates endothelial activation and lung injury. (*A* and *B*) Interventional Tz treatment of HUVECs post inflammatory insult with LPS, Il1β or TNFα, followed by qPCR for pro-inflammatory adhesion receptor *Vcam1* and chemokine *MCP1* (*A*) or immunoblot for Vcam1 (*B*). Graph: quantitation of Vcam1 normalized to GAPDH. (*C*) Schematic of interventional Tz treatment of animals injected with LPS that causes inflammatory lung injury. Lung sections were stained with H&E or CD68 to assess tissue morphology and inflammation marked by infiltrated immune cells (seven lungs from N = 4 male animals). Graph: quantitation of CD68+ cells per field of view. (*D* and *E*) Assessment of endothelial inflammation upon LPS injection, with or without Tz injection at 8 h. Graphs: quantitation of endothelial Vcam1 and nuclear Klf4 MFI (N = 3 male animals). Values are means ± SEM. Statistical analysis used one-way ANOVA (*A* and *B*) or unpaired two-tailed Student’s *t* test (*C*–*E*). [Scale bar, (*C*–*E*) 100 μm.]

We next considered whether Tz could limit inflammatory gene expression using an in vitro “therapeutic” protocol where it is added to already activated ECs. For this experiment, HUVECs were treated with or without Il1β, TNFα or LPS for 8 h, then Tz added at subsequent times. Induction of VCAM1, a leukocyte adhesion receptor, and MCP1 (CCL2), an inflammatory cytokine implicated in atherosclerosis, were strongly suppressed by later addition Tz (Fig. 6*A*). These effects correlated with strong upregulation of PRC2-catalyzed H3K27me3 levels and blockade by Tz, signifying strong positive correlation of PRC2 activity in response to diverse vascular or general inflammatory stimuli (Fig. 6*B*).

To explore the role of PRC2 in inflammation in vivo, we examined bacterial LPS, a potent inflammatory agonist that triggers EC activation and vascular leak, which, if severe, can induce septic shock and/or acute lung injury, mimicking acute respiratory distress syndrome (ARDS) (46). Mice were injected with LPS, resulting in the expected structural damage to lung tissue and infiltration of CD68+ macrophages/monocytes (Fig. 6*C*). Treatment with Tz at 8 h after LPS injection, at which point inflammation was established, reduced vascular inflammation and leukocyte infiltration 16 h later (Fig. 6 *C*–*E*). Thus, blocking PRC2/EZH2 ameliorates established endothelial inflammatory activation and limits tissue damage in this model.

### Therapeutic Intervention with Tazemetostat Alleviates Established ASCVD.

**Fig. 7.**
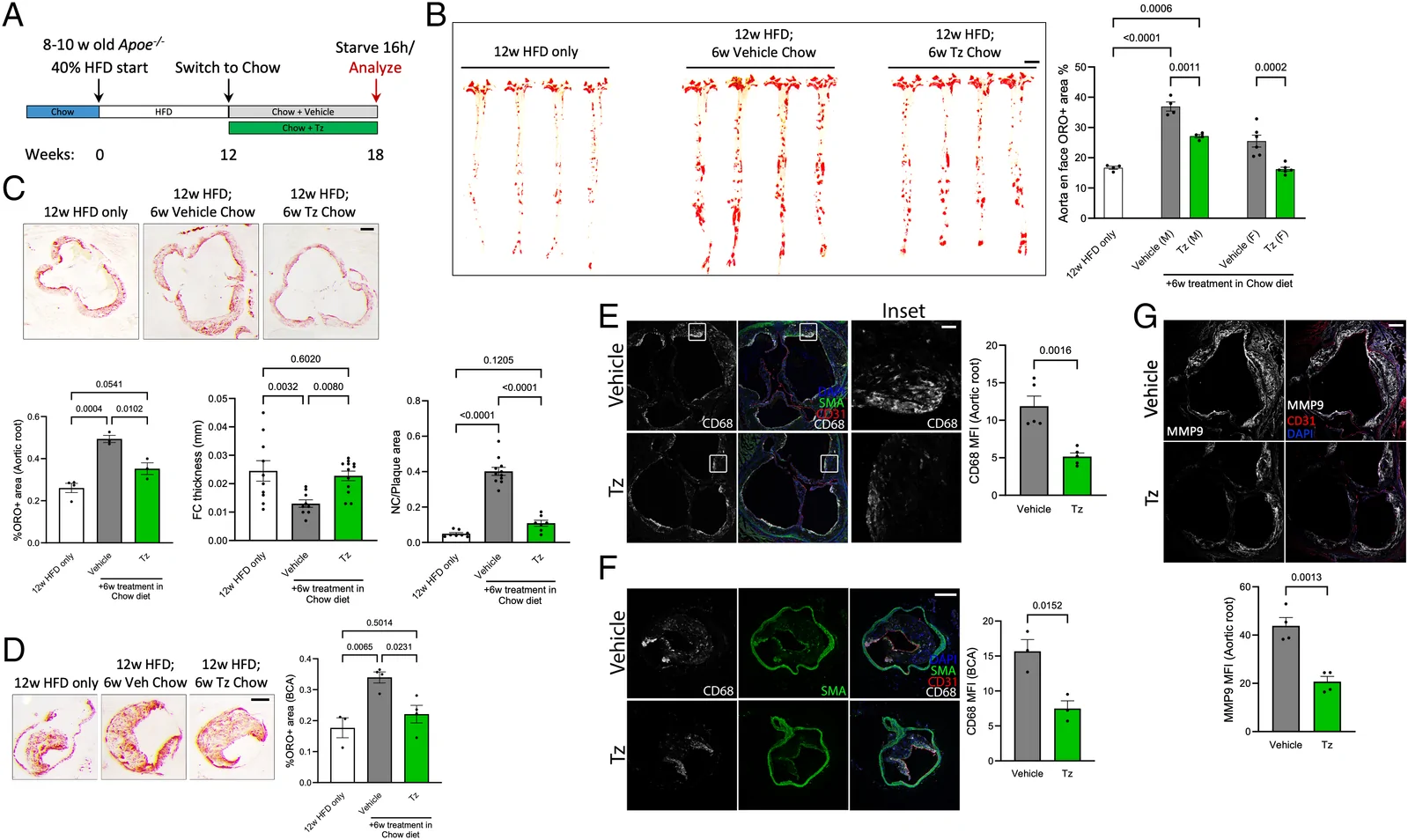
Therapeutic intervention with Tazemetostat alleviates pathology of established ASCVD. (*A*) Schematic of the experimental design. 8 to 10-wk-old *Apoe^−/−^* mice were fed a HFD for 12 wk, then switched to chow diet containing vehicle or Tz for an additional 6 wk. (*B*) Whole aortas were stained with Oil Red O to mark lipid-rich regions and imaged en face (N = 4 males and 6 females per condition). Graph: quantitation of percent Oil Red O positive area. (*C* and *D*) Sections from aortic roots (*C*) and BCA (*D*) were stained with Oil Red O (N = 3 animals). Graphs: quantitation of atherosclerotic plaque area (per animal), necrotic core (NC) area (per plaque), and fibrous cap (FC) thickness (per plaque). (*E* and *F*) Atherosclerotic plaques in aortic root sections (*E*) and BCA sections (*F*) were stained for the macrophage/monocyte marker CD68, and counterstained with SMA and DAPI to mark SMCs and nuclei respectively (N = 3 animals). Graph: quantitation of CD68 MFI. (*G*) Aortic root sections were stained for plaque vulnerability marker MMP9, and counterstained with CD31 and DAPI to mark ECs and nuclei respectively (N = 4 animals). Graph: quantitation of MMP9 MFI. Values are means ± SEM. Statistical analysis used one-way ANOVA (B–D) or unpaired two-tailed Student’s *t* test (E–G). [Scale bar, (*B*) 500 μm, (*C* and *D*) 100 μm, (*E*–*G*) 200 μm.]

These results prompted us to explore Tz in ASCVD. We examined Tz in a mouse model of ASCVD using an interventional strategy to mimic clinical practice (47, 48). *Apoe^−/−^* mice were maintained on a high fat diet (HFD) for 12 wk to induce hypercholesterolemia and ASCVD, then switched to chow diet with or without Tz. Switching to chow diet decreases circulating cholesterol from ~1,200 to ~300 mg/dL, while plaques progress more slowly (49). Vehicle or Tz was then administered in food for six additional weeks (Fig. 7*A*). Tz treatment slowed plaque growth by >50% (Fig. 7*B*). Importantly, plaques at the aortic root and brachiocephalic artery (BCA) showed ~2× thicker fibrous caps despite smaller plaque size and essentially no expansion of necrotic cores (Fig. 7 *C* and *D* and *SI Appendix*, Fig. S6*A*). Plaques in Tz treated mice also showed a >2× reduction in CD68+ monocyte/macrophages (Fig. 7 *E* and *F*) and similarly reduced MMP9 levels (Fig. 7*G*). In Tz treated animals, atherosclerosis-associated circulating inflammatory factors IFNγ and IL-2, and CCL3 showed trending reduction without reaching statistical significance (*SI Appendix*, Fig. S6 *B*–*D*). Body weight and circulating lipids (TAGs and cholesterol) were similar between vehicle and Tz treated cohorts, with the expected reduction in cholesterol after switching to chow diet (*SI Appendix*, Fig. S7 *A*–*C*). Tz intervention thus moderately slowed plaque growth but dramatically reduced markers of plaque vulnerability (50). These data confirm PRC2/EZH2 as a pro-inflammatory mediator in ASCVD and suggest possible use of Tz or other safe PRC2 inhibitors for treating advanced ASCVD and other vascular inflammatory disorders.

## Discussion

Endothelial inflammation underlies not only ASCVD (7, 9) but additional cardiovascular disorders including pulmonary arterial hypertension (51), acute lung injury (52), and vascular dementia (53), thus, is of major relevance to human health. Three independent bioinformatic approaches pointed to PRC2 as a positive regulator of endothelial inflammatory activation. Previous studies had implicated PRC2 in inflammation and ASCVD (16, 23, 25, 27–31, 54), but mechanistic understanding was limited and its role in established disease remained unexplored. We therefore looked further into mechanisms and efficacy of its inhibitor Tz in a preclinical model.

Deeper analysis by cSTAR of the network governing EC state transitions identified two main mechanisms driving the OSS state. The first ASCVD driver is cell cycle-dependent and comprises a cluster of mutually activating modules (CDK1, Aurora kinase, PAK, and ERK) stimulated by PRC2. The second driver consists of mutually activating TGFβ and BRD modules that are also activated by PRC2/EZH2 but distinct from cell cycle regulation. Consequently, suppression of PRC2/EZH2 inhibits two distinct ASCVD drivers. The inferred network further indicates that both of these pathways suppress the protective Klf2/4 module.

We confirmed that PRC2/EZH2 directly suppressed transcription of Klf2/4 in ECs by maintaining Histone H3 lysine 27 trimethylation (H3K27me3) on their promoters. Consistent with Klf2/4 suppression, the EZH2 inhibitor Tz reversed inflammatory gene expression after treatment with cytokines or LPS in vitro and in vivo, while in hyperlipidemic mice it slowed the increase in plaque size, and, of greater clinical importance, drastically improved plaque phenotype. Multiple in vitro and in vivo assays thus support an inflammatory role for PRC2/EZH2 in ECs and suggest that its inhibition may be useful for treating advanced vascular inflammatory disorders.

PRC2 is best known for preserving the pluripotent state of stem cells and cancer cells by suppressing genes that promote differentiation (55). Whether or how pro-inflammatory effects in ECs relate to this function is an open question. Studies of developmental and postnatal angiogenesis showed that arterial ECs are the last to form, originating from capillary and/or venous lineages, with no movement in the opposite direction (56–58). We suggest that this arterial EC state is comparatively resistant to inflammatory activation and leukocyte recruitment. This notion is consistent with postcapillary venous endothelium as the main site of physiological leukocyte trafficking into tissues (59). Klf2 expression is substantially higher in arterial ECs compared to capillary or venous (60, 61). Reanalysis of published scRNAseq data from mouse arteries shows an inverse correlation between expression of inflammatory genes and arterial identity genes (45) (*SI Appendix*, Fig. S2 *C* and *D*). PRC2 functions in these two settings may therefore have common elements.

The core catalytic subunit of PRC2 comprises either EZH1 or EZH2 methyltransferase. Compared to EZH2, EZH1 has lower catalytic activity and is also expressed at lower levels in most cell types (62). Nevertheless, both EZH1 and EZH2 may have important and redundant or disparate functions in endothelial biology. Our study focused specifically on PRC2/EZH2 as the major methyltransferase in ECs. For this reason, we use Tz throughout our study, which inhibits EZH2 with >35-fold selectivity relative to EZH1. Tz treatment almost completely eliminates H3K27me3 (Fig. 6*B* and *SI Appendix*, Fig. S2*C*), confirming the functional relevance of EZH2 as the major PRC2 methyltransferase in ECs.

Regulation of Klf2/4 via PRC2 implies that PRC2/EZH2 inhibition should boost Klf2/4 expression and protect from ASCVD. PRC2 is a general epigenetic suppressor of gene transcription that directly or indirectly targets thousands of genes (63). While several studies showed the involvement of PRC2/EZH2 in vascular inflammation and ASCVD (reviewed in ref. 20), the present study defines mechanisms of action and strengthens clinical relevance. Importantly, most cardiovascular events are caused by plaque rupture, which correlates with the vulnerable plaque phenotype rather than plaque size (64–66). We therefore utilized a therapeutic protocol in which *Apoe^−/−^* mice on HFD for 12 wk with preexisting disease were transferred to a chow diet to mimic lipid lowering therapy, plus the EZH2 inhibitor Tz. Tz moderately slowed the rate of plaque growth (~50%). This modest reduction is not unexpected given that mice remain hyperlipidemic, with circulating cholesterol around 300 mg/dL. But Tz intervention potently improved fibrous cap thickness, necrotic core size, and immune cell content, markers of plaque vulnerability to rupture, which is the key determinant of cardiovascular events.

Tz was FDA-approved for long term treatment of hematopoietic cancers with activated EZH2 (ClinicalTrials.gov ID NCT02875548). In that setting, patients treated with Tz over an 8-y period showed no increase in deaths from other causes compared to placebo. However, while this manuscript was under review, new data showing a modest increase in other hematopoietic cancers in Tz-treated patients led to voluntary withdrawal of Tz by the manufacturer (Ipsen). Other EZH2 inhibitors such as GSK343 and Tulmimetostat may circumvent this problem, alternatively, Tz may be useful for short term treatment of cardiovascular disease.

In summary, we conclude that PRC2 is a component of the endothelial inflammatory program, playing a key role in suppressing expression of the anti-inflammatory Klf2/4 genes, in part by direct modification of their promoters. Inhibiting the catalytic activity of EZH2 in a mouse model of atherosclerosis results in modest effects on plaque size but dramatic improvement of plaque phenotype, which is the major determinant of clinical events.

Major limitations of this study include general limitations of hyperlipidemic mice to model human disease, the precise composition of PRC2 in ECs, regulation of gene targets in ECs other than Klf2/4, and effects of Tz on additional cell types. While important for function, we have not determined the composition, assembly, and function of PRC2 complexes in ECs under different biomechanical states, as PRC2 can have a variable core and highly variable accessory factors. Future studies that comprehensively examine transcriptomic and epi-genomic states of cells in Tz-treated *Apoe^−/−^* atherosclerotic tissues could identify additional downstream target genes and clarify its role in other relevant cell types. For this last point, the increase in PRC2/EZH2 expression and activity in plaque ECs is notably greater than in immune cells, while it is reduced rather than increased in plaque SMCs (22, 67). Nonetheless, it may play significant functional roles in these cell types. More detailed understanding of the molecular mechanisms governing the regulation of PRC2 itself, the complex assembly and activity under biomechanical stimuli such as diverse shear stress profiles, PRC2-dependent epigenomic and transcriptional regulation, and effects in vascular inflammatory diseases are key areas for future work.

## Materials and Methods

Detailed methods are provided in *SI Appendix*. Cells were obtained from the Yale Vascular Biology and Therapeutics Core and ATCC. Shear stress experiments were performed in parallel plate flow chambers perfused with a pump in an environmental control system as described (68). Computational methods and analysis utilized the cSTAR pipeline described previously (3). All animal procedures followed Yale Environmental Health and Safety (EHS) regulations and were approved by Yale Institutional Animal Care and Use Committee (IACUC) and Yale Animal Resource Center (YARC). Methods on IHC, cell and molecular Biology, quantification, and statistics are described in *SI Appendix*.

### ARRIVE Reporting Guidelines.

Samples were randomly allocated into experimental groups. Age and sex-matched animals were randomly assigned to groups. Imaging fields were randomly selected. Experiments were blinded. Animal based experiments required two or more authors for simultaneous processing.

## Supplementary Material

Appendix 01 (PDF)

Dataset S01 (XLSX)

Dataset S02 (XLSX)

## Data Availability

Data generated and/or analyzed during this study are freely available. Resources generated during this study are either freely available or available from the corresponding author upon reasonable request. The RNAseq data (GEO accession number GSE343885) (69) and the code will be released upon publication. All other data are included in the manuscript and/or supporting information.
