# Supplementary material for: Polycomb Repressive Complex 2 drives flow-sensitive endothelial states underlying vascular inflammation and disease
Source: Proc Natl Acad Sci U S A. 2026 Aug 31;123(36):e2603888123. doi: 10.1073/pnas.2603888123 (PMC13552879; doi:10.1073/pnas.2603888123)
Supplement: Supplementary file 1 [file pnas.2603888123.sapp.pdf]

Polycomb Repressive Complex 2 drives flow-sensitive endothelial states underlying vascular inflammation and disease

Divyesh Joshi[1], Oleksii Rukhlenko[2], Raja Chakraborty[1], Anna Tuliakova[2], Zhongwei Sun[1], Tejas Bhogale[1#], Jessica Furtado[1,3], Hanqiang Deng[1], James G. Traylor Jr.[4], Hiroaki Imoto[2], Wayne Orr[4], George Tellides[5], Kathleen A. Martin[1], Boris Kholodenko[2,6], and Martin A. Schwartz[1,7,8,*]

Materials and methods

Primary cells, cell lines, and cell culture reagents

HUVECs pooled from 3 donors were obtained from the Yale Vascular Biology and Therapeutics Core. HAECs were obtained from ATCC. Cultures were screened for the absence of pathogens including mycoplasma, maintained in EGM2 Endothelial Cell Growth Medium (Lonza, CC-3162) and used at passages 2-5. SiRNA transfection was performed with Lipofectamine RNAiMAX (ThermoFisher, 13778150) in Opti-MEM medium (ThermoFisher, 31985070) using ON-TARGET plus SMARTpool siRNAs from Horizon Discovery (Dharmacon). For monocyte adhesion assays, THP-1 cells labeled with CellTracker Deep Red (ThermoFisher, C34565) in HBSS supplemented with 1 mM Ca2+, 0.5 mM Mg2+, and 0.5% BSA were added to the HUVECs, incubated for 20 min at 37°C, washed 3x in HBSS and fixed with 3.7% formaldehyde. Cells were counterstained with DAPI before fluorescence imaging. Treatments used were Tazemetostat (Selleckchem), GSK343 (Medchem, HY-13500), Tulmimetostat (Medchem, HY-145602), Flavopiridol Hydrochloride or Flav-HCl (Medchem, HY-10006), TNFα (PeproTech, 300-01A), IL-1β (Medchem, HY-P7028), and LPS from Escherichia coli O111:B4 (Sigma-Aldrich, 437627). All antibodies were validated in knockdown/knockout depletion by immunofluorescence and/or immunoblotting. Antibodies are listed in Table S1 and siRNAs in Table S2.

Shear stress

Shear stress experiments were performed in parallel plate flow chambers and perfused with a pump in environmental control system as described[1]. Briefly, cells were seeded at 70-90% confluency on 10 µg/ml Fibronectin-coated glass slides for 48-72 h in complete medium. For shear stimulation, slides were mounted in custom-made 25x55 mm parallel plate shear chambers with 0.5 mm thick silicone gaskets and stimulated with magnitudes of shear stress for LSS, PSS, and HSS as described, or 1 ± 4 dynes/cm^2 for OSS in complete media. The media was maintained at 37°C and 5% CO_2 with a heat gun and humidified bubbler, respectively.

Computational methods and analysis

This study utilizes the cSTAR pipeline described previously. Whereas the originally published pipeline[2], requires module activities to be measured directly through immediate downstream effectors, this subsequent cSTAR pipeline[3] infers protein activities via transcriptional signatures to construct causative networks.

Following the methodology established in our previous work[3], we utilized in-house HUVEC transcriptomics data under varying fluid shear stress (FSS) conditions to generate the State Transition Vectors (STVs). The STVs that govern FSS sensing of ECs were built using Support Vector Machine algorithm (SVM), separating transcriptomic states corresponding to different FSS levels. The STVs are unit vectors that are normal vectors to the hyperplanes separating distinct EC states. To build the STV_{FSS}, which describes the direction of vessel remodeling, we separated LSS and HSS states. STV_{remod}, which describes the initiation of vessel

remodeling program regardless of the inward or outward direction, was obtained by separation of LSS and HSS states from PSS state. To generate the STV_{OSS}, which describes pathological vessel remodeling, we separated PSS, LSS, and HSS states from OSS state. Mutual orthogonality of these STVs suggests that these three processes activate different groups of genes. The generated STVs are presented in the Dataset S2.

To identify key proteins governing EC transitions along these STVs, we used publicly available perturbation data, together with in-house generated transcriptomics data. In addition to CMAP dataset (LINCS L1000)[4] we used the following two publicly available RNAseq datasets: (i) perturbation of NOTCH pathway[5,6], and (ii) perturbation of KLF2/4[7,8]. In-house generated RNA-seq data were used for PRC2 module perturbations (Tazemetostat treatment). Analyzing the changes in the transcriptome, following drug perturbations targeting specific proteins, we identify protein modules, which are key components of regulatory networks controlling EC state transitions[3].

The first modification to previously published cSTAR pipeline[3] was augmentation of RNAseq data, performed as follows. First, differentially expressed genes were determined using DESeq2 R package, with $\boldsymbol{\mu}$ denoting vector of log2 fold changes (log2FoldChange column of DESeq2 output), and D denoting diagonal matrix of standard errors of log2 fold changes (lfcSE column of DESeq2 output). Next, Pearson correlation matrix P was calculated for log-normalized counts. Then, covariance matrix Σ of log2 fold changes of gene expression was estimated as

$$\Sigma = D \cdot P \cdot D \tag{1}$$

As a final step, augmented data (vectors of log2 fold changes) were generated by sampling multivariate Gaussian distribution with vector of mean values $\boldsymbol{\mu}$ and covariance matrix Σ, and only statistically significant differentially expressed genes were taken from augmented data. Each of RNAseq datasets used yielded exactly 1 vector of log2 fold changes, and each of such vectors was augmented to generate 20 vectors of augmented data. Next, we selected from RNAseq data only the genes that were directly measured (the so-called landmark genes) in L1000 assay. Then, both augmented RNAseq and L1000 data were merged in one table of log2 fold changes with respect to appropriate control, and served as an input for the next steps.

Using methods described in our previous work[3], we inferred the HUVEC-specific transcriptional signatures of selected protein modules, termed the 'module activity coefficients'. In brief, a relative activity of the of the i-th module after j-th perturbation (f_{ij}) is calculated as a linear function of the transcriptomic LFCs (x_{kj}) as follows,

$$f_{ij} = \tilde{\alpha}_{i0} + \sum_{k} \tilde{\alpha}_{ik} x_{kj} \tag{2}$$

Coefficients $\tilde{\alpha}_{ik}$, which are used to infer activity of i-th module, can be found by minimizing the following function[3]

$$F(\tilde{\alpha}_{ik}) = \tilde{a}\sum_{i}\left(\sum_{j_i}\left(\frac{1}{1+\frac{I_{j_i}}{K_{j_i}}} - 1 - \sum_{k}\tilde{\alpha}_{ik}x_{kj_i}\right)\right)^2 + \tilde{b}\sum_{i_1\neq i_2}\frac{\left(\sum_k \tilde{\alpha}_{i_1k}\cdot\tilde{\alpha}_{i_2k}\right)^2}{\sum_k \tilde{\alpha}_{i_1k}^2\cdot\sum_k \tilde{\alpha}_{i_2k}^2} + \tilde{c}\sum_{f_{ij}<0}\left|f_{ij}\right| + \tilde{d}\sum_{i,j}\left|\tilde{\alpha}_{ij}\right| \quad (3)$$

Here I_{j_i} are inhibitor doses doses and K_{j_i} are IC50 doses of inhibitors. When coefficients $\tilde{\alpha}_{ik}$ are determined, the elements of the global response matrix R_{ij} for core network modules can be calculated from Eq. 2 as follows

$$R_{ij} = 2\frac{f_{ij}-1}{f_{ij}+1} \quad (4)$$

Equations 2-4 allow us to quantify drug-induced changes in module activities and construct a global response matrix. This matrix served as the primary input for Modular Response Analysis (MRA)[9] to infer the regulatory networks governing transitions between cell states.

The second modification was an MRA extension. In the MRA framework, the matrix of experimentally measured global responses, R is connected to the matrix of connection strength (local response matrix) r via following expression [doi: 10.1016/j.celrep.2021.109157]

$$\boldsymbol{R} = -\boldsymbol{r}^{-1}\cdot\boldsymbol{r_p} \quad (5)$$

Here $\boldsymbol{r_p}$ is a matrix of the initial, immediate perturbations of primary perturbation targets while all perturbations are not allowed to propagate through the network. Since the extent of initial perturbations is unknown while global perturbation responses have been measured, the previous MRA methods for inference of connections to module i (i-th row of matrix r) selected only perturbations that do not directly affect module i[9]. In this case, elements of i-th row of matrix r can be found from the following equations[9]

$$\sum_{k=1,k\neq i}^{n} R_{kj}\cdot r_{ik} = R_{ij} \quad (6)$$

However, such approach limits the amount of data used for network inference by excluding some perturbations. To overcome this limitation, we have developed a new method, called MRA-RP, which uses all the data for inference of each row of local response matrix. The method consists of two steps. At the first step, matrix $\boldsymbol{r_p}$ is inferred from Eq. (5) using elastic net regression, where only those elements of $\boldsymbol{r_p}$ are allowed to be non-zero, which correspond to directly perturbed module. At the second step, matrix $\boldsymbol{r_p}$ was used to obtain local response matrix from the following equation

$$\sum_{k=1,k\neq i}^{n} R_{kj}\cdot r_{ik} = R_{ij} - \rho_{ij} \quad (7)$$

Here ρ_{ij} are non-zero elements of matrix $\boldsymbol{r_p}$. For inference of matrix r, orthogonal distance regression (ODR) was used, which is equivalent to weighted total least squares MRA[9].

To improve stability and convergence of this iterative procedure, under-relaxation technique was used for $\boldsymbol{r_p}$ as follows

$$r_p^i = (1-\alpha)r_p^{i-1} + \alpha\cdot r_p^{i*} \quad (8)$$

Here r_p^i is the matrix that enters Eq. (7), r_p^{i*} is a matrix inferred from Eq. (5), and α is a coefficient of under-relaxation. The code implementing MRA-RP with ODR regression and under-relaxation will be made available following the acceptance of this manuscript.

In summary, the modified pipeline facilitates the data augmentation of key regulatory modules and more effectively leverages available perturbation data for network inference. These enhancements proved especially critical for the successful integration of RNA-seq datasets with L1000 assay data.

After inference of the matrix r, the matrix $-r^{-1}$ was used to predict global responses of each module in the network to the activation of specific module (see Eq. 5).

Animals and tissue preparation

All animal procedures followed Yale Environmental Health and Safety (EHS) regulations and were approved by Yale Institutional Animal Care and Use Committee (IACUC) and Yale Animal Resource Center (YARC). Mice were maintained in a light- and temperature-controlled environment with free access to food and water and all efforts were made to minimize animal suffering. Euthanasia was performed by overdose of inhaled isoflurane and death confirmed by subsequent cervical dislocation and/or removal of vital organs and/or opening the chest cavity verified by the absence of cardiovascular function. Mice were perfused through the left ventricle with PBS and then 3.7% formaldehyde followed by tissue collection, as described[10]. The lungs, heart and spinal column, with aorta and carotids attached, were removed, fixed under gentle agitation for an additional 24 h at 4 °C, and washed 3x with PBS. For whole aorta *en face* prep, the isolated aortas were bisected along the lesser curvature and the aortic arch was bisected through the greater curvature as well. For aortic arch segment preparation, the aortic arch was bisected through the greater curvature. Hearts (containing aortic roots), carotids and lungs were allowed to sink in 30% sucrose in PBS overnight at 4°C, embedded in OCT (optimal cutting temperature compound, Sakura, 4583) and frozen on dry ice before sectioning. Tissue blocks were cut into 8-10 µm sections using a cryostat (Leica) and sections stored at -80°C until use.

Lipopolysaccharide (LPS) lung injury

To induce acute lung inflammation, 8-12-week-old male C57BL/6J mice received a single intraperitoneal (i.p.) dose of lipopolysaccharide (LPS from Escherichia coli O111:B4, Sigma-Aldrich) at a dose of 1 mg/kg body weight, diluted in sterile PBS to a final volume of 100 µL. 8 hours post LPS, mice received a single i.p. dose of Tz at a dose of 150 mg/kg body weight. Control animals received an equal volume of vehicle. Mice were euthanized 24 hours post LPS injection for tissue collection.

Atherosclerosis progression and blood lipid analysis

To induce atherosclerosis, 8-12-week-old male and female *Apoe*$^{-/-}$ mice were maintained on a high-fat diet (HFD; Clinton/Cybulsky high-fat rodent diet with regular casein and 1.25% added cholesterol; Research Diet, D12108c) for 12 weeks. Mice were then switched to chow diet containing either vehicle or Tazemetostat (Tz) at 150 mg/kg/day for a further 6 weeks. Mice after 12 weeks HFD were sacrificed before switching to chow diet and Tz treatment to establish the atherosclerotic burden prior to treatment. Tz amounts were calculated considering 3g food intake per mouse per day. Blood samples from overnight starved mice were collected in EDTA-coated tubes, centrifuged at 8,000g at 4°C for 10 min and the supernatant (plasma) was separated and stored at -80 °C. Total plasma cholesterol and triglycerides (TAG) were measured using kits according to the manufacturer's instructions (Wako Pure Chemicals, Japan). Tissues were prepared and analyzed as described below.

Tissue analysis

For IF, OCT tissue sections were thawed and washed 3x with PBS to remove OCT. Cells were fixed with 3.7% formaldehyde for 15 min at ambient temperature, washed with and stored in PBS. Deidentified human specimens were deparaffinized in Histo-Clear (National Diagnostics, HS-200). Sections were progressively rehydrated before antigen retrieval for 30 min at 95°C in 1X Antigen Retrieval Buffer (Dako, 51699). Samples were incubated in perm-block buffer (5% donkey serum, 0.2% BSA, 0.3% Triton X-100 in PBS) for 1 h at room temperature, incubated with primary antibodies in perm-block overnight at 4°C, washed three times in perm-block and then incubated with alexafluor-conjugated secondary antibodies (ThermoFisher) at 1:1000 dilution in perm-block for 1 h at room temperature. Slides were washed 3x in perm-block and 3x in PBS before mounting in DAPI Fluoromount-G (Southern Biotech; 0100–20). Images were acquired on a Leica SP8 confocal microscope equipped with the Leica Application Suite software. Confocal stacks were flattened by maximum intensity z-projection in ImageJ. Post background subtraction, the mean fluorescence intensity (MFI) or nuclear intensity (with DAPI mask) was recorded. For aorta ORO staining, the whole aorta was opened longitudinally on a soft-bottomed silica dish, incubated with 0.6% ORO in 60% isopropanol with gentle rocking for 1 h at ambient temperature, washed in 60% isopropanol for 20 minutes, washed in dH2O 3x, and mounted on slides with endothelium side up in OCT compound. Images were acquired with a digital microscopic camera (Leica DFC295). ORO staining on OCT tissue sections was done similarly. Quantitation of ORO positive area was done in ImageJ. Hematoxylin and eosin (H&E) staining on OCT tissue sections was done by Yale Research Histology Core using standard techniques. Plaque morphometric and vulnerability analysis was performed as described[10]. Plaque area was determined by ORO positive staining. For each plaque, the necrotic core (NC) area was defined as a clear area in the plaque that was H&E free, and the fibrous cap (FC) thickness was quantified by selecting the largest necrotic core and measuring the thinnest part of the cap.

Immunoblotting

Cells were lysed in RIPA buffer (Roche) containing 1x Halt Protease Inhibitor Cocktail (ThermoFisher, 78429) and 1x PhosStop (Roche, 4906837001) for 30 min on ice, clarified at 13,000 g 4°C for 15 minutes, the supernatant transferred to new 1.5 ml tubes, 4x loading buffer (250mM Tris-HCl pH 6.8, 8% SDS, 40% glycerol, 20% β-mercaptoethanol, 0.008% bromophenol blue) added and the samples heated to 95°C for 5 min. Samples were resolved by a 4-15% gradient SDS Polyacrylamide gel, and transferred to 0.2 µm nitrocellulose membranes. Nitrocellulose was blocked with 5% non-fat skim milk for 1h at ambient temperature, incubated with desired antibodies diluted in 5% BSA using a standard immunoblotting procedure and detection by ECL (Millipore). Images were quantified in ImageJ by densitometry and normalized to GAPDH or Tubulin loading controls.

RNA isolation, sequencing, and Chromatin Immuno-precipitation (ChIP), ChIP quantitative real-time PCR (ChIP-qPCR)

Total RNA was extracted from cells using the RNeasy Plus Mini Kit (Qiagen, 74136) according to the manufacturer's instructions. RNA was quantified by NanoDrop, and RNA integrity was measured with an Agilent Bioanalyzer. Samples were sequenced using Illumina NovaSeq 6000 (HiSeq paired-end, 100 bp). The base calling data from the sequencer were transferred into FASTQ files using bcl2fastq2 conversion software (version 2.20, Illumina). Published datasets with identifiers GSE276195 and GSE272071 were reanalyzed. Three replicates per condition were used. PartekFlow (a start-to-finish software analysis solution for next generation sequencing data applications) was used for analysis. Raw data in FASTQ format were assessed for quality using FastQC. Adapter sequences and low-quality bases were trimmed from the reads using fastp with default parameters. The resulting high-quality reads were aligned to the human reference genome (GRCh38) using STAR aligner. Differential gene expression (DEG) analysis was performed using DESeq2 (median of ratios normalized) using significance thresholds of $|\log2(\text{fold change})| > 1$ and

p-value < 0.05. Transcription factor and upstream regulator analysis was conducted using EnrichR and X2KWeb, where DEGs from transcriptomics analysis were used as inputs to identify upstream regulators and pathways. ChIP-qPCR primers are listed in Table S3.

Quantification and Statistical Analysis

Morphometric analysis was done with ImageJ software (version 1.51; National Institutes of Health, Bethesda, MD). Graph preparation and statistical analysis was performed using GraphPad Prism 10.0 software (GraphPad software Inc.). Data with less than 30 replicates were tested for normality using Shapiro-Wilk test (Figs 2c,d,e, 4c,d,g, 5d, 6a,b,c,d, 7b,c,d,e,f,g, S1c,d,e, S5, S6a,b,c). Data with 30 or more replicates were considered normally distributed (Figs 4e, 5a,b,c,f,g, 6e). Statistical significance was performed using Student t test for two group comparison or one-way ANOVA with Tukey's post hoc analysis for multiple groups comparison, as described in the figure legends. Data are presented as means ± SEM. A p value less than 0.05 was considered significant.

Supplementary figure legends

Figure S1. RNAseq analysis. (a) Heat map showing log_2fold-change (FC) of selected shear-sensitive genes from bulk RNAseq of HUVECs exposed to PSS vs OSS. (b) X2KWeb analysis to identify common upstream regulators of DEGs from bulk RNAseq analysis of Control si, Pcdhg si and Pcdhg+Klf2/4 triple si HUVECs. Enriched upstream regulators from Fig 1e were connected by X2KWeb using an expanded protein-protein interaction network. Enriched regulators are in red, while the proteins that connect them are in grey. The size of the nodes is proportional to their degree of enrichment. (c) Reanalysis of published RNAseq dataset (GSE272071) showing mRNA levels (normalized read counts) of Ezh2 and Suz12 after Pcdhg depletion. (d,e) Immunoblot for Ezh2, Suz12 and Klf4 in Control or Pcdhg si HUVECs under physiological laminar shear stress (PSS) (N=3 independent experiments). Graph: quantitation of Ezh2, Suz12, and Klf4 normalized to GAPDH loading control.

Figure S2. Expression of Ezh1 and Ezh2 in vascular cells. (a, b) Analysis of mRNA levels of Ezh1 and Ezh2 from human RNA atlas for multiple vascular cell types. (c) Functional validation of Ezh2 si and Suz12 si in HUVECs exposed to OSS and stained for H3K27me3. (d) Immunoblot for pro-inflammatory adhesion receptor VCAM1 in HUVECs, untreated or treated with LPS for 24 h with or without Tz. Scale bar: (c) 20 µm.

Figure S3. ChIPseq analysis. (a) Klf2 promoter region showing peaks for H3K27ac (marking its promoter) and H3K27me3 from ChIPseq analysis of HUVECs (UCSC Genome Browser). (b) H3K27me3 ChIPseq analysis of control si and Pcdhg si HUVECs showing a significantly higher peak call on the Klf2 promoter (N=3 independent experiments). (c) FUCCI-HAECs were incubated for 24 hours with or without Tz and the percentages of Late vs Early G1 cells were quantified (N=3 independent experiments, n>1000 cells/experiment). Values are means ± SEM. Statistical analysis used unpaired two-tailed Student's t-test (c, d).

Figure S4. PRC2 is associated with CVD. (a) Human coronary artery sections from de-identified elderly donors stained for H3K27me3 and leukocyte common antigen CD45, comparing regions of plaque (Plaque) to the adjacent regions of the same artery without evident plaque (Control). Sections were counterstained with DAPI to mark nuclei (N=3 donors). (b-d) Re-analysis of Ezh2, Suz12 and Pcdhg in major plaque cell types including ECs, immune cells and SMCs (b) from published scRNAseq data from deidentified human ASCVD patients comparing regions of plaque (Plaque) to an adjacent plaque-free region of the same artery

(Normal)[11]. TNFα and Klf2/4 serve as internal controls for plaque ECs and normal ECs respectively (N=3 donors). (c) Feature plot showing an inverse correlation between the expression of arterial marker Efnb2 and the inflammatory markers Mmp2, Selp and Ccl2 in plaque ECs. (d) Heat map showing Efnb2 levels in Mmp2^{low} (<0.5) vs Mmp2^{high} (>1.5) in plaque ECs from three individual specimens.

Figure S5. Pharmacological inhibition of PRC2 increases KLF4 protein. (a) Pharmacological inhibition of PRC2/EZH2 with Tz in HUVECs under static (St) or PSS, immunoblotted for Klf4 and GAPDH loading control (N=3 independent experiments). Graph: quantitation of Klf4 normalized to GAPDH. (b) PRC2/EZH2 inhibition with GSK343 and Tulmimetostat (Tulmi) in HUVECs under PSS, immunoblotted for Klf4 and GAPDH loading control (N=3 independent experiments). Graph: quantitation of Klf4 normalized to GAPDH. (c) Validation of PRC2/EZH2 inhibition by Tz, GSK343, and Tulmimetostat in HUVECs by immunostaining for H3K27me3. Graph: quantitation of H3K27me3 MFI. (d) PRC2/EZH2 inhibition with Tz, GSK343, and Tulmi in HAECs under PSS, immunoblotted for Klf4 and GAPDH loading control. Values are means ± SEM. Statistical analysis used one-way ANOVA.

Figure S6. Tz in treating established ASCVD. (a) Sections from brachiocephalic artery (BCA) from atherosclerotic mice treated with or without Tz (as in Fig 7), stained with H&E (N=3 animals). Graphs: quantitation of necrotic core (NC) area (per plaque) and fibrous cap (FC) thickness (per plaque). (b-d) Analysis of circulating cytokine/chemokine levels, as indicated. Values are means ± SEM. Statistical analysis used unpaired two-tailed Student's t-test (a-d).

Figure S7. Mouse weight and blood lipid analysis. (a-c) Body weights (a), plasma triglycerides (TAGs) and cholesterol content after overnight starvation, mouse groups indicated (N = 5). Values are means ± SEM. Statistical analysis used one-way ANOVA.

Supplementary Data Table S1. List of primary antibodies.

Antibody	Company, Catalog No.	Dilution for IF	Dilution for immunoblot
H3K27me3	Cell Signaling, 9733	1:800	1:2000
H3K27me3	Abcam, ab6002	1:500	1:2000
Klf4	Abcam, ab215036	1:500	1:4000
H3 total	Cell Signaling, 4499	-	1:2000
Vcam1	Abcam, ab134047	-	1:4000
Klf2	ProSci, 16-624	-	1:2000
GAPDH	Cell Signaling, 5174	-	1:8000
Ezh2	Cell Signaling, 5246	-	1:2000
Suz12	Cell Signaling, 3737	-	1:2000
MMP9	Abcam, ab58803	1:400	-
CD68	ThermoFisher Scientific, 14-0681-82	1:200	-
CD31	R&D Systems, AF3628	1:200	-
SMA	Millipore Sigma, A2547	1:500	-
Erg	Abcam, ab92513	1:800	-
CD45	R&D Systems, AF114	1:200	-

Supplementary Data Table S2. List of siRNAs.

Gene sg/si	Details
Hs Pcdhg si#1	Custom si, Horizon Discovery, CCGCCCAACACGGACTGGCGTTT
Hs Pcdhg si#2	Custom si, Horizon Discovery, CCCAACAACCAGTTTGACACAGA
Hs Klf2 si	Horizon Discovery, L-006928-00-0005
Hs Klf4 si	Horizon Discovery, L-005089-00-0005
Hs Suz12 si	Horizon Discovery, L-006957-00-0005
Hs Ezh2 si	Horizon Discovery, L-004218-00-0005

Supplementary Data Table S3. Hs *Klf2* promoter ChIP-qPCR primers.

	Sequence (5'->3')	Strand	Length	Start	Stop	Tm	GC%
Forward primer	TCCCATCCATCCAGGGTTCT	Plus	20	15	34	59.95	55
Reverse primer	TCAGAGACTCTCAGGGGAGC	Minus	20	124	105	60.03	60
Product length	110						

References

1. Conway, D. E. *et al.* VE-Cadherin Phosphorylation Regulates Endothelial Fluid Shear Stress Responses through the Polarity Protein LGN. *Curr Biol* **27**, 2727 (2017). https://doi.org/10.1016/j.cub.2017.08.064
2. Rukhlenko, O. S. *et al.* Control of cell state transitions. *Nature* **609**, 975-985 (2022). https://doi.org/10.1038/s41586-022-05194-y
3. Deng, H. *et al.* cSTAR analysis identifies endothelial cell cycle as a key regulator of flow-dependent artery remodeling. *Sci Adv* **11**, eado9970 (2025). https://doi.org/10.1126/sciadv.ado9970
4. Subramanian, A. *et al.* A Next Generation Connectivity Map: L1000 Platform and the First 1,000,000 Profiles. *Cell* **171**, 1437-1452 e1417 (2017). https://doi.org/10.1016/j.cell.2017.10.049
5. Fang, J. S. *et al.* Shear-induced Notch-Cx37-p27 axis arrests endothelial cell cycle to enable arterial specification. *Nat Commun* **8**, 2149 (2017). https://doi.org/10.1038/s41467-017-01742-7
6. Souilhol, C. *et al.* JAG1-NOTCH4 mechanosensing drives atherosclerosis. *Sci Adv* **8**, eabo7958 (2022). https://doi.org/10.1126/sciadv.abo7958
7. Sangwung, P. *et al.* KLF2 and KLF4 control endothelial identity and vascular integrity. *JCI Insight* **2**, e91700 (2017). https://doi.org/10.1172/jci.insight.91700
8. Sweet, D. R. *et al.* Kruppel-Like Factors Orchestrate Endothelial Gene Expression Through Redundant and Non-Redundant Enhancer Networks. *J Am Heart Assoc* **12**, e024303 (2023). https://doi.org/10.1161/JAHA.121.024303
9. Santra, T., Rukhlenko, O., Zhernovkov, V. & Kholodenko, B. N. Reconstructing static and dynamic models of signaling pathways using Modular Response Analysis. *Current Opinion in Systems Biology* **9**, 11-21 (2018). https://doi.org/https://doi.org/10.1016/j.coisb.2018.02.003
10. Joshi, D. *et al.* Endothelial gamma-protocadherins inhibit KLF2 and KLF4 to promote atherosclerosis. *Nat Cardiovasc Res* **3**, 1035-1048 (2024). https://doi.org/10.1038/s44161-024-00522-z
11. Alsaigh, T., Evans, D., Frankel, D. & Torkamani, A. Decoding the transcriptome of calcified atherosclerotic plaque at single-cell resolution. *Commun Biol* **5**, 1084 (2022). https://doi.org/10.1038/s42003-022-04056-7
